# Improving emotion regulation and mood in teacher trainees: Effectiveness of two mindfulness trainings

**DOI:** 10.1002/brb3.1390

**Published:** 2019-08-22

**Authors:** Lena Wimmer, Lisa von Stockhausen, Silja Bellingrath

**Affiliations:** ^1^ School of Psychology University of Kent Canterbury UK; ^2^ Department of Psychology University of Duisburg‐Essen Essen Germany

**Keywords:** emotion regulation, mindfulness, mood, teacher trainees

## Abstract

**Background/Objective:**

The present research investigated potential effects of mindfulness training on emotion regulation and mood of future schoolteachers in a nonrandomized pre–post design, and whether these are influenced by the yoga component of mindfulness‐based stress reduction (MBSR) and/or by homework practice.

**Method:**

*N* = 169 university students received either mindfulness training (experimental groups), awareness activities (active control group), or no training (passive control group), in the context of university seminars. Allocation to groups was bound by the seminar chosen by participants, and in that sense was self‐selected. Mindfulness was trained in two adapted MBSR courses, one of which including yoga, and the other excluding yoga.

**Results:**

Specific benefits of both mindfulness training groups were observed for emotion regulation in terms of an increase in cognitive reappraisal and a reduction in symptom‐focused rumination as well as depressive mood. No benefits of mindfulness training were observed for reductions in expressive suppression, self‐focused rumination, anxious, and negative mood or an increase in distraction and positive mood respectively. Mindfulness training with and without yoga was mostly equally effective. Outcomes were largely not moderated by practice quantity or quality, but reductions in depressive mood were mediated by gains in reappraisal and distraction.

**Conclusions:**

Mindfulness training can be implemented in the context of university seminars to foster advantageous emotion regulation strategies and lower depressive mood in future schoolteachers. Discontinuing yoga within mindfulness interventions does not seem to reduce training benefits.

## 1 INTRODUCTION

Emotion regulation (ER), defined as “attempts individuals make to influence which emotions they have, when they have them, and how these emotions are experienced and expressed” (Gross, Richards, & John, 2006, p. 14), is an inherent part of everyday life. On one hand, successful ER is considered a requirement of adaptive functioning (Gross et al., 2006) that often draws on strategies such as cognitive reappraisal, in terms of thinking of a situation differently in order to change its emotional impact (Gross & John, 2003), distraction, in terms of purposely directing attention to pleasant or neutral activities (Nolen‐Hoeksema, Wisco, & Lyubomirsky, 2008) as well as acceptance, in terms of the ability to remain in contact with feelings, thoughts, and physical sensations without attempting to change them (Aldao & Nolen‐Hoeksema, 2012). On the other hand, inefficient ER, which is frequently associated with strategies such as suppression of emotional expression (Gross & John, 2003), ruminative and repetitive thinking (Nolen‐Hoeksema et al., 2008), or the avoidance of situations, thoughts or sensations that elicit unpleasant affect (Aldao & Nolen‐Hoeksema, 2012), can lead to various clinical symptoms, such as panic attacks, binge eating, and substance abuse (Sheppes, Suri, & Gross, 2015).

Schoolteachers are among the professions with particularly high demand on ER skills, which are necessary for the successful management of challenging student behavior and for coping with their own emotional states (Skinner & Beers, 2016). Inadequate ER can result in clinical levels of anxiety and depression in teachers, and in adverse effects on students via reduced quality of instruction (McLean & Connor, 2015).

Incorporating training of ER skills into university education seems therefore necessary to prepare future teachers for their job requirements. Furthermore, most mental health difficulties begin at the age during which most future teachers study at university (Reavley, 2018), which is why higher education institutions have been encouraged to be an environment for interventions with a preventive effect on mental health and well‐being issues (Galante et al., 2018). Similarly, ER has generally been found to shift toward an increasingly adaptive pattern throughout adulthood, with young adults being inferior to older adults (Zimmermann & Iwanski, 2014), particularly regarding ER strategies that do not primarily draw on executive functioning (EF; Consedine & Mauss, 2014). These age differences may in part go back to changes in structural factors of the work environment: when compared to typical work environments in later adulthood, work settings of young adults may more often demand expressive suppression (John & Gross, 2004), and are characterized by a high density of stressful events as well as elevated levels of job insecurity (Scheibe & Zacher, 2013).

For teacher trainees, a paradigmatic situation requiring suppression is when candidates try not to show disappointment after receiving negative feedback on a demonstration lesson from a supervisor. Further specific emotional challenges can arise from preparing new subject matter; planning lessons; acquiring classroom management; building relationships with colleagues, supervisors, pupils, and parents; coping with constant assessment; and financial problems (Rieg, Paquette, & Chen, 2007; Totterdell & Parkinson, 1999).

Taken together, training of ER in teacher trainees would not only prepare attendees for future job demands, but it would also reduce heightened risk of mental health problems, bolster natural improvement of ER strategies involving relatively little executive control, and counteract age‐related decrement of those ER strategies that recruit relatively much EF.

Over the past decades, mindfulness training has been identified as an effective strategy for improving mental health and well‐being, including reductions in anxious and depressive mood (Hofmann, Sawyer, Witt, & Oh, 2010). Mindfulness is often defined as awareness arising from a nonjudgmental present‐moment attention focus (Kabat‐Zinn, 2003). A recent meta‐analysis (Gu, Strauss, Bond, & Cavanagh, 2015) demonstrated that mindfulness‐related benefits for well‐being may be due to the acquisition of more functional ER strategies. Furthermore, there also is direct evidence for a positive effect of mindfulness practice on ER skills (Roemer, Williston, & Rollins, 2015), however, the underlying mechanisms of a mindful ER have not been fully understood (Hölzel et al., 2011). For instance, there is an ongoing debate as to whether mindful ER involves top‐down processes relying on voluntary executive control, such as reappraisal (e.g., Farb, Anderson, Irving, & Segal, 2014; Garland, Hanley, Goldin, & Gross, 2017; Hölzel et al., 2011), or whether it recruits bottom‐up processes that mainly target bodily representations of emotional states, such as perceptual sensations (e.g., Chambers, Gullone, & Allen, 2009). At present, it seems most integrative to assume that mindful ER entails a complex set of strategies comprising both top‐down and bottom‐up processes (Guendelman, Medeiros, & Rampes, 2017). One could speculate that individuals starting their mindfulness practice rely more on effortful top‐down processes and, with increasing experience, gradually shift toward more frequent use of bottom‐up processes (Chiesa, Serretti, & Jakobsen, 2013), either because attentional processes become automated (Chambers et al., 2009), or because experts deal differently with emotions in the sense that they let go of appraisals (Hölzel et al., 2011).

According to initial empirical investigations, mindfulness training can be considered a promising solution to improve teachers' ER skills and mood (overviews: Emerson et al., 2017; Hwang, Bartlett, Greben, & Hand, 2017; Klingbeil & Renshaw, 2018; Lomas, Medina, Ivtzan, Rupprecht, & Eiroa‐Orosa, 2017), with potential downstream effects on classroom performance. In their systematic review of mindfulness‐based interventions for schoolteachers, Emerson et al. (2017) considered a range of outcomes and suggested the greatest potential for ER. However, in the systematic review by Lomas et al. (2017), only three out of 19 studies reported on ER, with most studies looking at rather distal outcomes such as stress and burnout, but not at whether these effects are linked with more adaptive ER skills.

For instance, in two randomized controlled trials (RCTs; Roeser et al., 2013), teachers that received mindfulness training demonstrated greater focused attention, working memory capacity, mindfulness, and occupational self‐compassion, as well as lower occupational stress and burnout at both postprogram and 3‐month follow‐up, when compared to participants from the control group. In a randomized pilot trial of a modified mindfulness‐based stress reduction (MBSR) course (Flook, Goldberg, Pinger, Bonus, & Davidson, 2013), intervention‐related improvements in self‐compassion and performance on a computer‐based task of affective attentional bias, and reductions in psychological symptoms and burnout were found and accompanied by increases of observer‐rated classroom organization. Similarly, in a RCT investigating the Cultivating Awareness and Resilience in Education (CARE for Teachers) program, Jennings, Frank, Snowberg, Coccia, and Greenberg (2013) observed benefits of this mindfulness‐based course on well‐being, burnout/time‐related stress, mindfulness, and teaching efficacy. To sum up, although existent findings indicate benefits of mindfulness‐based interventions for teacher well‐being and efficacy, more research is needed to explore potential underlying effects of enhanced ER. This applies to a greater extent to teacher trainees, a target group for which the evidence base lags behind.

Following this, it should be investigated how to tailor mindfulness training to the context of university education, the intervention setting for teacher trainees. Based on the general mindfulness literature and specific challenges of the field setting of universities, the following aspects appear particularly worthwhile:

First, assessing the contribution of individual program elements and exclusion of the least effective element(s). Mindfulness‐based interventions are typically multicomponent programs. MBSR, the best‐known mindfulness‐based intervention, utilizes three core exercises, namely: breathing meditation, body scan, and yoga/mindful movement (Baer & Krietemeyer, 2006). While breathing meditationand body scan are considered formal meditation exercises where practitioners aim to focus their attention on a single object, that is, one's breath in breathing meditation and certain parts of one's body in the body scan, yoga exercises emphasize bodily movements/postures (Schmalzl, Powers, & Blom, 2015). Regarding feasibility, yoga exercises seem more challenging to implement in the setting of a university seminar than meditation, due to the configuration and size of classrooms. Breathing meditation and body scan can easily be practiced while seated at a desk, whereas many yoga exercises require more space for which furniture needs to be rearranged or removed. It would therefore be worthwhile to test whether discontinuing yoga exercises within MBSR is associated with fewer benefits for ER than MBSR including yoga. Although advantageous effects on ER have been observed for both mindfulness‐based interventions (see above) and yoga as a stand‐alone treatment (Menezes et al., 2015), they can be expected to be stronger for mindfulness training than yoga: Mindfulness training is primarily a mental activity, which is expanded by a movement component in yoga (Schmalzl et al., 2015), so that part of the mental resources consumed during yoga need to be spent on correct execution of positions. Consequently, these resources are lost for immediate ER. The partition of mental capacity is reflected in main practice intentions as reported by practitioners (especially among beginners), namely alleviation of emotional stress for mindfulness (Pepping, Walters, Davis, & O'Donovan, 2016), and both exercise and stress relief for yoga (Park, Riley, Bedesin, & Stewart, 2016).

A second way to tailor interventions (to teacher trainees) is to examine dose–response effects and implement the lowest dose that is required to yield a certain result. A crucial method to control the dose of mindfulness training needed is via assigning varying amounts of homework practice. A recent meta‐analysis demonstrated small to moderate associations between the extent of homework practice and outcomes of mindfulness training (Parsons, Crane, Parsons, Fjorback, & Kuyken, 2017). The authors suggest considering further participant engagement variables, such as quality of homework practice (Vettese, Toneatto, Stea, Nguyen, & Wang, 2009), so that the relationship between training and outcomes can be more fully explained.

The present study investigated the effects of mindfulness training on ER skills and mood of teacher trainees. To facilitate the implementation of a mindfulness program for this target group, two adapted MBSR courses were compared, one of them including, the other one excluding yoga exercises. Comparable effects of both programs would suggest that the inclusion of yoga is not necessary to strengthen ER strategies or to reduce anxious/depressive mood in future teachers. In order to determine whether a certain amount and/or quality of homework practice is necessary to achieve benefits, participants were asked to keep logs. Effects unspecific to mindfulness training were controlled for by an active and a passive control group. The active control group engaged in activities that explored the phenomenology of awareness and were based on Blackmore and Troscianko (2018). Similar to mindfulness training, these activities required meta‐cognition. However, in contrast to mindfulness training, the awareness activities did not instruct participants to regulate their emotions or mood at all. Thus, the active control group controlled for unspecific meta‐cognitive processes. To control for test–retest effects, the influence of intermittent events and education at university, the passive control group attended regular classes at university only.

The following hypotheses were tested:

1. Both mindfulness training groups show greater benefits in ER strategies as well as stronger improvements in mood than the passive control group, whereas the active control group does not demonstrate such benefits.
2. Mindfulness training without yoga leads to comparable benefits in ER strategies and mood as mindfulness training with yoga.
3. Mindfulness‐based effects on mood are mediated by changes in ER.
4. Mindfulness‐based effects on ER and mood are moderated by amount and quality of homework practice.

## 2 METHOD

The study followed a nonrandomized pre–post design with two experimental groups (mindfulness training with yoga, mindfulness training without yoga), an active (awareness activities) and a passive control group.

### 2.1 Participants

**Figure 1:**
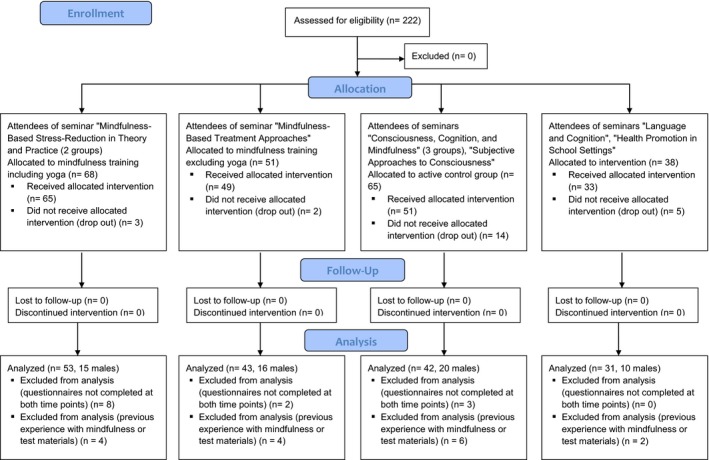
Flow of participants through the study

Two hundred and twenty‐two university students were recruited in nine psychology classes held at the University of Duisburg‐Essen between October 2015 and July 2016 (spanning two semesters). All attendees of these nine seminars were considered eligible for research participation. The sample size reflects the number of students that could be trained by qualified staff in the period given, and therefore, is based on available resources in terms of personnel. Allocation to the training and control groups was based on the classes which the students attended, and in that sense happened by way of self‐selection. However, students were only informed about the study in the first seminar session and could choose to attend the class without participating in the study. Volunteers received course credits for participation. The flow of participants is shown in Figure 1. The final sample consisted of *N* = 169, with *n* = 53 participants (15 of them male) in the mindfulness including yoga group, *n* = 43 participants (16 male) in the mindfulness excluding yoga group, *n* = 42 participants (20 male) in the awareness activity group, and *n* = 31 participants in the passive control group (10 male). The mean age of the 163 participants who indicated their age was 24.87 years (*SD* = 3.46).

Analyses using G*Power revealed that the total sample size of *n* = 169 had a power of >0.99 to detect a global effect in a MANOVA, a power of >0.99 to detect mediation, and a power of 0.97 to detect moderation, when the standard 5% significance level and a medium effect size (*f* ^2^ = 0.15) were applied (see results section below for details on these inferential statistics).

### 2.2 Interventions

All interventions were embedded into regular psychology classes at the University of Duisburg‐Essen, Germany, with students intending to become schoolteachers being the target audience. Treatment groups were instructed by the authors of the present paper, who had engaged in mindfulness practice over several years. The first author taught one seminar receiving mindfulness training excluding yoga and one of the seminars for the awareness activity. The second author led three seminars receiving the awareness activity and one seminar for the passive control group. The third author gave two classes for the mindfulness training group including yoga and one class that contributed to the passive control group. The fact that the interventions were incorporated into seminars on mindfulness/consciousness theory restricted the possibility to blind participants. However, no information about study aims or hypotheses was given to participants before post‐tests were finished.

#### 2.2.1 Mindfulness trainings

Mindfulness‐based stress reduction (Kabat‐Zinn, 1990) served as a basis of the mindfulness trainings. The university setting required the following adaptations of the course structure: the original yoga exercises were replaced by office yoga poses (Meyer, 2013; for the training group including yoga); the sessions lasted 1.5 hr during which participants received theoretical input about mindfulness (about 45 min) as well as a mindfulness training comprising of the contents of MBSR including practice (about 45 min); the fourth session was used to discuss practice experiences and ways of coping with difficulties; the day of mindfulness was dropped (for the schedule of each session cf. Table 1). One introductory session and seven training sessions took place biweekly. Participants were asked to do 20 min of formal homework exercises (alternating between body scan and breathing meditation and—in one group—yoga) on, at least, 5 days of the week, and were provided with taped audio instructions. Informal practices were not assigned because they are difficult to quantify, which conflicts with the aim to investigate the effect of practice duration and frequency. Both mindfulness groups were assigned the same amount of homework, which included body scan and breathing meditation in the nonyoga condition and body scan, breathing meditation, and yoga in the yoga condition.

**Table 1:** Schedule of the mindfulness trainings

Session number	MBSR input	Theoretical input
1	Becoming acquainted with mindfulness	Modeling of mindfulness
2	Perception and meditation	Effects of mindfulness practice: Empirical evidence
3	Exploring boundaries	Mindfulness‐based interventions: Dialectical behavior therapy and acceptance and commitment therapy
4	Reflection of mindfulness practice	–
5	Mindfulness‐based stress reduction	Area of application: Schools
6	Intensive mindfulness practice/mindful communication	–
7	Taking care of oneself/maintaining practice	Area of application: Interpersonal relationships

Abbreviation: MBSR, mindfulness‐based stress reduction.

#### 2.2.2 Control groups

The design of the study included an active and a passive control group. To allow for identifying specific effects of mindfulness training, the active control group received phenomenologically oriented awareness activities adopted from Blackmore and Troscianko (2018), which controlled for effects of unspecific meta‐cognitive processes. During these exercises, participants reflected on their current state of consciousness by asking themselves questions such as “Am I conscious now?”. Homework consisted of reflecting on one of these questions as often as possible without any instruction to regulate thoughts or emotions. The awareness activity was practiced out of class only, though participants' experiences and possible difficulties were discussed in class.

In order to control for effects of repeated assessment, intermittent events, and education at university, the passive control group did not receive any training at all.

### 2.3 Instruments

#### 2.3.1 Emotion regulation

Two self‐report questionnaires served as indicators of ER. First, we used a German version (Abler & Kessler, 2009) of the Emotion Regulation Questionnaire (ERQ) by Gross and John (2003), where participants report on levels of reappraisal and suppression by responding to 10 items on a seven‐point rating scale. In the context of this instrument, reappraisal is considered a beneficial ER strategy, whereas suppression is regarded as an ER strategy detrimental to well‐being.

The second measure of ER was the German 23‐item version of Nolen‐Hoeksema's (1991) Response Style Questionnaire (RSQ), the RSQ‐D (Kühner, Huffziger, & Nolen‐Hoeksema, 2007). Participants indicate on a 5‐point rating scale how often they cope with sad or depressed mood using (a) symptom‐focused rumination, (b) self‐focused rumination, and (c) distraction. According to Response Style Theory (Nolen‐Hoeksema et al., 2008), rumination maintains or exacerbates depressed mood, whereas distraction alleviates it.

#### 2.3.2 Mood

Participants reported on their general mood using the German version (Breyer & Bluemke, 2016) of the Positive and Negative Affect Schedule (PANAS; Watson, Clark, & Tellegen, 1988). The PANAS is a widely employed mood scale. Respondents rated 10 positive and 10 negative affect adjectives on a 5‐point scale as descriptors of their mood during the last week. Two scores are derived which represent negative and positive affect, respectively.

Anxious and depressive moods were operationalized via the German version (Herrmann‐Lingen, Buss, & Snaith, 2011) of the Hospital Anxiety and Depression Scale (Zigmond & Snaith, 1983), the HADS‐D. In this questionnaire, the extent of anxious and depressive symptoms over the previous week is self‐reported on two respective subscales. Fourteen items are to be rated on a 4‐point scale.

#### 2.3.3 Practice properties

Quantity of homework in all treatment groups (mindfulness training with yoga, mindfulness training without yoga, awareness activities) was assessed by utilizing self‐report diaries that were filled in at home. Each diary covered a period of 2 weeks, except for one diary that additionally included a 2‐week holiday at the end of the year. Quality of mindfulness practice was recorded once per diary, immediately after the last exercise of each practice period, via a German translation of the Toronto Mindfulness Scale (TMS; Lau et al., 2006). The TMS is a self‐report questionnaire measuring state mindfulness via 13 items that are to be rated on a 5‐point scale. As such, it does not seem suited to operationalize quality of homework in the awareness activity group. Nevertheless, participants of this group completed the TMS to avoid biases between the mindfulness training and the awareness practice groups that are related to reactivity in response to measuring instruments, such as effects of position and sequence. In addition, practice quality was measured via open responses, which are not reported in this article.

Adverse events were not explicitly monitored.

### 2.4 Procedure

The study was approved by the Ethics Committee of the Department of Psychology, University of Duisburg‐Essen and conformed to the Declaration of Helsinki. All participants provided written‐informed consent and received course credit for participation.

Participants filled in all self‐report questionnaires except the ones assessing practice properties at the experimental laboratory of the Language & Cognition Unit at the Department of Psychology, University of Duisburg‐Essen, at the beginning and the end of the semester. Respondents were provided with the following order of questionnaires: RSQ, ERQ, HADS‐D, PANAS. Examiners were student research assistants who were blind to participants' treatment conditions. Interventions started after the pretests had been completed and continued for a whole semester, that is, 12 weeks. The post‐tests were conducted immediately after the interventions were completed.

### 2.5 Data analysis

Statistics were conducted using SPSS, employing the standard *p* < .05 significance level. Individuals were the smallest unit of analysis. As outlined in Figure 1, participants were excluded from analyses if they (a) dropped out from the respective seminar, (b) did not complete the questionnaires at both time points, or (c) reported previous experience with mindfulness and/or had participated in a previous study using the same dependent measures. Missing questionnaire items were replaced with the series mean, using the respective function in SPSS. For the ERQ, data of one additional participant could not be analyzed due to insufficient completion at pretest.

## 3 RESULTS

### 3.1 Effects on ER and mood

Analyses of variance for each of the dependent pretest measures as well as age revealed baseline differences between the four groups for RSQ, symptom‐focused rumination, *F*(3, 165) = 6.00, *p* = .001, RSQ, self‐focused rumination, *F*(3, 165) = 8.82, *p* < .0001, and PANAS, negative affect, *F*(3, 165) = 3.80, *p* = .011 (other *p*s* *> .13). LSD post hoc tests showed that the awareness activity group scored higher on symptom‐focused rumination, self‐focused rumination, and negative affect than all other groups at baseline (symptom‐focused rumination: *p*s < .003; self‐focused rumination: *p*s < .0002, negative affect: *p*s < .030). According to a contingency analysis, groups did not differ concerning gender, *p* = .258.

Descriptive statistics and reliabilities for dependent measures, as observed in the current sample, are displayed in Table 2.

**Table 2:** Descriptive statistics and internal consistencies of dependent measures by experimental conditions and times of testing

	Cronbach's alpha		Passive control group		Awareness activity (Active control group)		Mindfulness training without Yoga		Mindfulness training with Yoga	
	T1	T2	T1: *M* (*SD*)	T2: *M* (*SD*)	T1: *M* (*SD*)	T2: *M* (*SD*)	T1: *M* (*SD*)	T2: *M* (*SD*)	T1: *M* (*SD*)	T2: *M* (*SD*)
ERQ—reappraisal	0.81	0.85	26.35 (6.93)	24.45 (8.69)	25.54 (7.94)	23.79 (7.53)	24.96 (6.61)	26.43 (6.82)	24.58 (6.16)	25.96 (5.42)
ERQ—suppression	0.71	0.73	13.45 (5.18)	13.03 (5.43)	15.27 (5.45)	15.83 (6.27)	14.28 (5.17)	12.89 (5.09)	12.77 (5.44)	12.40 (5.50)
RSQ—symptom‐focused rumination	0.78	0.78	11.97 (4.31)	13.96 (4.96)	15.86 (5.82)	16.17 (5.93)	12.65 (3.23)	12.81 (3.67)	13.05 (3.94)	13.08 (3.72)
RSQ—self‐focused rumination	0.79	0.77	12.68 (3.98)	12.64 (3.53)	17.41 (5.88)	16.95 (5.69)	12.98 (4.108)	12.28 (3.67)	13.458 (4.59)	13.51 (4.36)
RSQ—distraction	0.75	0.75	21.81 (5.77)	20.94 (6.75)	20.21 (6.09)	20.36 (5.58)	19.12 (4.76)	23.30 (5.94)	19.84 (4.99)	21.51 (5.68)
PANAS—positive affect	0.85	0.89	33.68 (8.27)	31.10 (9.54)	32.60 (6.57)	31.50 (6.58)	33.13 (5.69)	32.77 (7.70)	33.48 (7.21)	33.30 (7.27)
PANAS—negative affect	0.87	0.87	18.12 (5.57)	20.10 (8.87)	21.83 (8.86)	21.90 (7.85)	17.10 (5.03)	17.17 (4.92)	18.77 (6.66)	18.62 (6.58)
HADS‐D—anxiety	0.80	0.81	6.03 (4.67)	6.68 (5.27)	7.52 (4.46)	7.52 (4.31)	5.98 (3.87)	5.72 (3.49)	6.49 (3.59)	6.32 (3.48)
HADS‐D—depression	0.78	0.85	3.19 (3.68)	4.10 (4.34)	4.45 (3.33)	4.90 (4.29)	3.19 (3.20)	2.65 (2.82)	3.27 (2.97)	3.06 (2.89)

Abbreviations: ERQ, Emotion Regulation Questionnaire; HADS‐D, Hospital Anxiety and Depression Scale; PANAS, Positive and Negative Affect Schedule; RSQ, Response Style Questionnaire; T1, pretest; T2, post‐test.

Hypothesis 1 regarding mindfulness‐specific benefits for ER strategies and mood was tested in a MANOVA with group (both mindfulness trainings collapsed, active and passive controls) as independent variable and difference scores between both time points as dependent variables. Pretest scores on RSQ, symptom‐ and self‐focused rumination, and PANAS, negative affect, were entered as covariates to account for baseline differences. The MANOVA, using Pillai's trace, demonstrated a significant effect of group. Follow‐up ANOVAs were significant for ERQ reappraisal, RSQ symptom‐focused rumination, RSQ distraction, and HADS‐D depression, (cf. Table 3; other *p*s* *> .07). Across all these effects, only the contrast comparing passive controls with mindfulness training reached significance, consistently demonstrating superior development of mindfulness training over no treatment.

**Table 3:** MANOVAs and follow‐up ANOVAs investigating the effect of mindfulness training on emotion regulation and mood

	Analyses across both mindfulness training groups	Analyses with separate consideration of both mindfulness training groups
MANOVA		
*V*	0.192	0.260
*F* (d*f*_M_, d*f*_R_)	1.83 (18, 310)	1.63 (27, 465)
*p*	.021	.025
Partial *η* ^2^	.096	.087
Follow‐up ANOVAs		
ERQ Reappraisal		
*F* (d*f*_M_, d*f*_R_)	4.998 (2, 166)	3.314 (3, 165)
*p*	.008	.021
Partial *η* ^2^	.057	.057
Contrast: passive controls versus active controls (95% CI, *p*)	[−2.925, 3.232], .922	[−2.934, 3.242], .922
Contrast: passive controls versus mindfulness (95% CI, *p*)	[0.634, 6.006], .016	N/A
Contrast: passive controls versus mindfulness excl. yoga (95% CI, *p*)	N/A	[0.296, 6.442], .032
Contrast: passive controls versus mindfulness incl. yoga (95% CI, *p*)	N/A	[0.332, 6.229], .029
RSQ Symptom‐focused rumination		
*F* (d*f*_M_, d*f*_R_)	3.689 (2, 165)	N/A
*p*	.027	.066
Partial *η* ^2^	.043	N/A
Contrast: passive controls versus active controls (95% CI, *p*)	[−2.255, 0.853], .374	N/A
Contrast: passive controls versus mindfulness (95% CI, *p*)	[−2.975, −0.364], .013	N/A
Contrast: passive controls versus mindfulness excl. yoga (95% CI, *p*)	N/A	N/A
Contrast: passive controls versus mindfulness excl. yoga (95% CI, *p*)	N/A	N/A
RSQ Distraction		
*F* (d*f*_M_, d*f*_R_)	8.222 (2, 166)	7.714
*p*	.0004	.00007
Partial *η* ^2^	.090	.123
Contrast: passive controls versus active controls (95% CI, *p*)	[−2.255, 0.853], .374	[−1.277, 3.320], .382
Contrast: passive controls versus mindfulness (95% CI, *p*)	[−2.975, −0.364], .013	N/A
Contrast: passive controls versus mindfulness excl. yoga (95% CI, *p*)	N/A	[2.758, 7.332], .00002
Contrast: passive controls versus mindfulness incl. yoga (95% CI, *p*)	N/A	[0.340, 4.730], .024
HADS‐D Depression		
*F* (d*f*_M_, d*f*_R_)	3.282 (2, 166)	N/A
*p*	.040	.080
Partial *η* ^2^	.038	N/A
Contrast: passive controls versus active controls (95% CI, *p*)	[−1.679, 0.777], .469	N/A
Contrast: passive controls versus mindfulness (95% CI, *p*)	[−2.333, −0.190], .021	N/A
Contrast: passive controls versus mindfulness excl. yoga (95% CI, *p*)	N/A	N/A
Contrast: passive controls versus mindfulness incl. yoga (95% CI, *p*)	N/A	N/A

Note

Follow‐up ANOVAs are reported only for outcomes that were significantly affected in at least one analysis.

Abbreviations: ERQ, Emotion Regulation Questionnaire; HADS‐D, Hospital Anxiety and Depression Scale; RSQ, Response Style Questionnaire.

To test hypothesis 2 regarding the comparability of mindfulness training including and excluding yoga, the two mindfulness training groups were treated as separate levels. Using Pillai's trace, the MANOVA detected a significant effect of group (cf. Table 3). The follow‐up ANOVAs were again significant for ERQ reappraisal, and RSQ distraction, yet the ANOVAs turned marginal for RSQ symptom‐focused rumination, and HADS‐D depression (other *p*s* *> .06). Regarding both significantly affected outcomes, the contrast of passive controls versus mindfulness training without yoga and the contrast of passive controls versus mindfulness training with yoga were the only significant contrasts.

To test whether the results were masked by the level of seminar which was partly confounded with the level of group (i.e., mindfulness without yoga was taught in one seminar, whereas mindfulness with yoga was taught in two seminars, awareness training was taught in four seminars, and the passive control group consisted of two seminars), a MANOVA was conducted with seminar (nine levels) as independent variable. This analysis, again using Pillai's trace, was insignificant, *p *= .066.

This was likewise applied regarding the teacher of each seminar that was also partly confounded with the level of group (see above). When using Pillai's trace, the MANOVA demonstrated a significant effect of teacher, *V *= 0.190, *F*(18, 310) = 1.811, *p *= .023, partial *η* ^2^ = .095. Follow‐up ANOVAs were significant for RSQ distraction only, *F*(2, 166) = 3.53, *p *= .0002, partial *η* ^2^ = .095 (other *p*s > .06). According to contrasts, the seminars taught by author 1 increased more strongly regarding distraction than the seminar taught by author 2, 95% CI for difference [−4.28, −0.80], *p *= .004, and author 3, 95% CI for difference [−6.25, −2.15], *p *= .00008.

### 3.2 Mediating impact of ER on the association of mindfulness training with mood

To test hypothesis 3 concerning mediating effects of ER, mediation analyses were carried out using the Process command for SPSS (model 4; Hayes, 2013). Both mindfulness trainings were pooled. Results are summarized in Table 4. In general, mediations were found for the mindfulness group only. Distraction mediated the relationship of mindfulness training with HADS‐D anxiety and HADS‐D depression. In addition, reappraisal acted as mediator in the relationship of mindfulness training with HADS‐D depression, and symptom‐focused rumination meditated the association of mindfulness training with HADS‐D anxiety.

**Table 4:** Models of treatment group (via mindfulness training, awareness activities, or no treatment) as predictor of mood, mediated by emotion regulation

Mediator	Outcome	Indirect effect	
		Contrast passive control group versus awareness activity	Contrast passive control group versus mindfulness training
ERQ reappraisal	PANAS negative affect	*b* = −.02, 95% CI [−0.45, 0.52]	*b* = −.39, 95% CI [−1.09, 0.04]
ERQ suppression		*b* = .04, 95% CI [−0.34, 0.41]	*b* = −.02, 95% CI [−0.38, 0.18]
RSQ symptom‐focused rumination		*b* = −.51, 95% CI [−1.73, 0.17]	*b* = −.57, 95% CI [−1.81, 0.10]
RSQ self‐focused rumination		*b* = −.010, 95% CI [−0.87, 0.35]	*b* = −.07, 95% CI [−0.73, 0.19]
RSQ distraction		*b* = −.16, 95% CI [−0.768, 0.25]	*b* = −.57, 95% CI [−1.30, 0.03]
ERQ reappraisal	PANAS positive affect	*b* = .03, 95% CI [−0.63, 0.72]	*b* = .58, 95% CI [−0.02, 1.47]
ERQ suppression		*b* = −.23, 95% CI [−0.93, 0.44]	*b* = .13, 95% CI [−0.40, 0.88]
RSQ symptom‐focused rumination		*b* = .20, 95% CI [−0.36, 1.18]	*b* = .122, 95% CI [−0.34, 1.30]
RSQ self‐focused rumination		*b* = .04, 95% CI [−0.25, 0.60]	*b* = .03, 95% CI [−0.13, 0.46]
RSQ distraction		*b* = .17, 95% CI [−0.28, 0.65]	*b* = .61, 95% CI [−0.12, 1.41]
ERQ reappraisal	HADS‐D anxiety	*b* = −.01, 95% CI [−0.27, 0.24]	*b* = −.120, 95% CI [−0.57, 0.04]
ERQ suppression		*b* = .05, 95% CI [−0.14, 0.29]	*b* = −.03, 95% CI [−0.26, 0.11]
RSQ symptom‐focused rumination		*b* = −.30, 95% CI [−0.91, 0.04]	*b* = −.34, 95% CI [−0.92, −0.01]
RSQ self‐focused rumination		*b* = −.08, 95% CI [−0.55, 0.31]	*b* = −.06, 95% CI [−0.44, 0.18]
RSQ distraction		*b* = −.11, 95% CI [−0.44, 0.15]	*b* = −.38, 95% CI [−0.83, −0.03]
ERQ reappraisal	HADS‐D depression	*b* = −.02, 95% CI [−0.42, 0.39]	*b* = −.37, 95% CI [−0.86, −0.04]
ERQ suppression		*b* = −.01, 95% CI [−0.26, 0.15]	*b* = .01, 95% CI [−0.16, 0.15]
RSQ symptom‐focused rumination		*b* = −.15, 95% CI [−0.72, 0.17]	*b* = −.16, 95% CI [−0.74, 0.16]
RSQ self‐focused rumination		*b* = −.02, 95% CI [−0.230, 0.19]	*b* = −.01, 95% CI [−0.26, 0.09]
RSQ distraction		*b* = −.12, 95% CI [−0.45, 0.18]	*b* = −.44, 95% CI [−0.90, −0.010]

Note

For mediator and outcome variables change scores, that is, the difference of post‐test–pretest each, were used. The confidence interval for the indirect effect is a bootstrapped CI based on 5,000 samples.

Abbreviations: ERQ, Emotion Regulation Questionnaire; HADS‐D, Hospital Anxiety and Depression Scale; PANAS, Positive and Negative Affect Schedule; RSQ, Response Style Questionnaire.

### 3.3 Moderating effects of homework quantity and quality

To test hypothesis 4 (mindfulness‐based effects on ER and mood are moderated by amount and quality of homework practice), two sets of moderation analyses were calculated using the Process command for SPSS (models 3/1; Hayes, 2013). In the first set, the average TMS sum score and the total duration of homework practice were included as moderators. The predictor variable was group (mindfulness training without yoga vs. mindfulness training with yoga). The awareness activity group was not included here because the TMS cannot be considered a valid measure of homework quality in this group. In the second set of moderations, total duration of homework practice was the only moderator. In return, the predictor variable group now included all three treatment groups.

Both sets of analyses yielded an interaction of practice duration with group for ERQ suppression, set 1: *b* = −.005, 95% CI [−0.010, −0.001], *SE B* = .002, *t* = −2.51, *p* = .014; set 2: *b* = −.005, 95% CI [−0.009, −0.001], *SE B* = .002, *t* = −2.39, *p* = .018, in that the mindfulness training group without yoga benefitted from increased practice duration, whereas the mindfulness training group with yoga did not. There were no further moderating effects of practice quality/quantity (*p*s > .07).

## 4 DISCUSSION/CONCLUSIONS

The present study is the first to investigate to what extent mindfulness training improves teacher trainees' ER skills. It also addressed the question of effective components of mindfulness training, underlying mechanisms of possible effects alongside the influence of duration and quality of practice. Two mindfulness trainings, one including and the other excluding yoga, were compared to test the necessity of MBSR's yoga component for achieving the anticipated benefits under investigation. Based on a relatively large sample, these two experimental groups were contrasted with both an active and a passive control group. Thus, it was not only possible to examine the contribution of yoga as a component of mindfulness‐based interventions but additionally, due to the design of the study, it was also possible to disentangle specific effects of mindfulness training, mindfulness‐unspecific but intervention‐related effects (in particular general meta‐cognitive processes), and intervention‐unrelated effects (e.g., test repetition and maturation). Furthermore, potential mediating effects of ER in the relationship between mindfulness training and improved mood, as well as potential moderating effects of quantity and quality of outside‐class practice were taken into account. This is valuable from both a theoretical and practical point of view; theoretically, mediation and moderation analyses permit insight into mindfulness‐related mechanisms of action, for instance, regarding the question whether mindfulness training in teacher trainees promotes well‐being via improvement of ER. Practically, such analyses can provide knowledge necessary to optimize the effectiveness of mindfulness‐based interventions, for example, as to whether instructors should encourage a certain level of practice quality, so that particular benefits can occur. Since the study was conducted in a real‐life setting where implementation is desired, that is, university seminars for teacher trainees, generalizability can be regarded as high.

Hypothesis 1 regarding specific benefits of mindfulness training on ER strategies and mood was mainly confirmed, as both mindfulness training groups consistently outperformed the passive control group in respect to reappraisal, symptom‐focused rumination, distraction, and depressive mood. Although no mindfulness‐based benefits were found for suppression, self‐focused rumination, general, and anxious mood, this hypothesis can be regarded as widely corroborated because superior development of the active controls over the passive controls was not observed at all. Hence, results of previous meta‐analyses, both for the general population and in‐service teachers (Emerson et al., 2017; Hofmann et al., 2010; Hwang et al., 2017; Klingbeil & Renshaw, 2018; Lomas et al., 2017), were largely replicated. It should, however, be noted that increases in distraction appeared to partially go back to teacher effects—groups taught by author 1 demonstrated higher increments than groups taught by the other authors, independent from the respective curriculum (mindfulness or awareness activities). Hence, benefits for distraction cannot be interpreted as entirely mindfulness‐specific but could be traced back to instruction methods unconsciously used by author 1.

Hypothesis 2 regarding effects of yoga as part of mindfulness training was confirmed: When the two mindfulness training groups were treated as separate treatments, results were comparable in so far as both groups showed increases of reappraisal and distraction and no further effects. Thus, one can conclude, from a practical point of view, that improving teacher trainees' ER and mood in a university setting can be achieved without the yoga element of MBSR. The result that both mindfulness trainings groups reported increased use of distraction attenuates the teacher effect on distraction mentioned above: The mindfulness training group including yoga was not taught by author 1 for whom this effect had been found. Since this group also demonstrated increments in distraction, effects related to author 1 cannot entirely account for changes in distraction.

Hypothesis 3 regarding mediation of mindfulness‐based effects on mood by changes in ER was partly confirmed. The only mediations observed were that in the mindfulness groups an increase in distraction mediated advantageous changes of most indicators of negative mood, namely decreases in anxiety, and depression (the former was also mediated by reductions in symptom‐focused rumination, while the latter was mediated by increases in reappraisal as well). Changes in suppression and self‐focused rumination did not act as mediators. Although more research is needed to better understand the mechanisms underlying mindfulness‐based benefits for well‐being, the current evidence can be regarded as consistent with previous results. This, in turn, can potentially suggest feasibility and fidelity of the adapted mindfulness curriculum under investigation.

Hypothesis 4 regarding possible moderation of mindfulness‐based effects on ER and mood by amount and quality of homework practice was mainly not confirmed. There was only one statistically significant moderation, whose practical importance can be considered questionable due to a small effect size. Thus, in the present study, the amount and quality of homework practices hardly affected the outcomes under investigation. This conflicts with a recent meta‐analysis (Parsons et al., 2017) that detected small to moderate associations between the amount of home practice and intervention outcomes. This could be explained by the fact that the studies included in the meta‐analysis differed from the present approach, both regarding curriculum length—8 weeks in the meta‐analysis versus 12 weeks in the present study and sample characteristics—clinical populations in the meta‐analysis versus general population in the current study.

### 4.1 Theoretical and practical implications

The present findings that mindfulness training for teacher trainees achieves benefits for ER and well‐being, and for well‐being via improved ER, are consistent with existing models and empirical evidence on mindfulness in general (e.g., Gu et al., 2015; Nykliček, 2011). Thus, mindfulness training in teacher trainees seems to work similarly as in other target groups.

In the mindfulness literature, there has been an ongoing debate as to whether mindful ER is either a top‐down (e.g., reappraisal) or bottom‐up (e.g., focusing on interoception) process. Most recently, it had been proposed that mindful ER involves a set of complex mechanisms, including both top‐down and bottom‐up processes (Guendelman et al., 2017), and that in the course of practitioners' mindfulness “career,” there might be a shift from an emphasis of top‐down processes toward bottom‐up processes (Chiesa et al., 2013). Since our sample included exclusively mindfulness‐inexperienced students and showed mindfulness‐related gains in reappraisal, the present results are in line with this notion. Whether, however, the sort of reappraisal used by participants relied on *effortful* top‐down regulation cannot be inferred from the current data. Nevertheless, since gains in reappraisal were not moderated by practice properties, the present findings do not support the view that mindfulness‐based improvements in reappraisal require regular training *over 12 weeks* (yet there might still be certain minimum training requirements below 12 weeks that we were unable to detect). Potentially, an increase in reappraisal stemmed from a change in individuals' attitude toward emotions that demands less training, such as realizing that emotions are not facts but associated with subjective appraisals.

Another open question in the mindfulness literature reflects on the contribution of individual MBSR components (e.g., Dimidjian & Linehan, 2003). According to the present results, the yoga component of MBSR does not seem to be irreplaceable by the combination of breathing meditation and body scan, assuming enhanced ER and well‐being are the desired outcomes.

The promising implication for teacher trainee development is that mindfulness training can be effectively embedded into university seminars to promote students' ER skills and well‐being. Such training would prepare teacher trainees for elevated demands on ER skills required in their future career for efficacious teaching and coping with their own emotions (McLean & Connor, 2015; Skinner & Beers, 2016). Furthermore, mindfulness training could prevent mental health difficulties frequently starting at student age (Reavley, 2018). There are two additional implications that facilitate implementation: first, according to the results, discontinuing the yoga element from MBSR is not associated with reduced benefits on ER or well‐being. This lowers demands on size/arrangement of classrooms since yoga exercises typically require more space than formal meditation practices, for which students could remain seated at their desks. Second, lacking moderations of practice quantity could imply that courses <12 weeks in length are sufficient to achieve gains in ER and well‐being. Shorter courses, in turn, would reduce time demands on both teaching staff and participating students.

### 4.2 Limitations

The following limitations restrict the explanatory power of the current approach. Firstly, we were not able to allocate participants randomly to conditions due to the context of the study (university), since seminars had to be freely selectable by students. The lack of randomization might have led to systematic group differences other than the interventions of interest. Although baseline differences were statistically controlled for, there could have been further biases; hence, differing group results cannot be attributed to intervention effects only. Secondly, because the interventions were embedded into university seminars, the requirements of practice duration had to be comparable for all participants so that individual variations were registered as they naturally occurred, rather than actively manipulated. This means that moderating effects of practice properties cannot be considered as causally linked with amount or quality of practice. Thirdly, the interventions were nested in several levels, and levels were partially confounded, which was inevitable due to shortness of qualified instructors. Again, this bias limits the possibility to link outcomes with interventions effects alone. However, follow‐up analyses showed that there was only a confounding effect of one of the nested levels, namely teacher, on one outcome variable, namely distraction. Fourthly, the fact that the interventions were delivered by the authors of this paper, who were involved in both study design and manuscript preparation, presents another potential bias. Even though teaching in the intervention classes was based on identical materials and followed a strict schedule, teaching styles, and knowledge of the hypotheses may have influenced results. Finally, imputing missing values by the series mean as in the present study has to be considered inferior to other strategies such as multiple imputation.

## 5 CONCLUSIONS

Emotion regulation is a critical skill for adaptive functioning in our daily lives. Because school teachers are faced with elevated emotional challenges in their career, training of efficient ER skills deserves to be integrated into teacher education. This would not only benefit teachers' mental health and well‐being, but potentially also improve the quality of instruction they provide to their pupils. The present evidence suggests that mindfulness training can be effectively incorporated into university seminars to equip future teachers with adaptive ER strategies that help reduce negative affect; these benefits can even be achieved even when yoga practice, a core element of MBSR, is not or cannot be implemented during training.

## CONFLICT OF INTEREST

None declared.

## Data Availability

The data that support the findings of this study are openly available in the Appendix S1 accompanying this paper.
